# Relationship between Diagnostic Method and Pathogen Detection, Healthcare Resource Use, and Cost in U.S. Adult Outpatients Treated for Acute Infectious Gastroenteritis

**DOI:** 10.1128/jcm.01628-22

**Published:** 2023-01-16

**Authors:** Rena C. Moon, Tammy C. Bleak, Ning A. Rosenthal, Brianne Couturier, Rachael Hemmert, Tristan T. Timbrook, Harold Brown, Ferric C. Fang

**Affiliations:** a PINC AI Applied Sciences, Premier Inc., Charlotte, North Carolina, USA; b Global Medical Affairs, bioMérieux, Inc., Salt Lake City, Utah, USA; c Department of Laboratory Medicine and Pathology, University of Washington, Seattle, Washington, USA; d Department of Microbiology, University of Washington, Seattle, Washington, USA; Maine Medical Center Department of Medicine

**Keywords:** acute infectious gastroenteritis, burden, diarrhea, healthcare cost, healthcare resource use, stool pathogen

## Abstract

A retrospective observational study was performed to assess the relationship between diagnostic method (traditional work-up [TW], multiplex PCR panel with < 12 target pathogens [PCR < 12], or multiplex PCR panel with ≥ 12 target pathogens [PCR12]), and diagnostic yield, health care resource use (HRU), and cost in adult outpatients visiting U.S. hospitals for acute infectious gastroenteritis (AGE). Using data from PINC AI Healthcare Database during January 1, 2016–June 30, 2021, we analyzed adult patients with an AGE diagnosis and stool testing performed during an outpatient visit. Detection rates for different pathogens were analyzed for those with microbiology data available. Among 36,787 patients, TW was most often performed (57.0%). PCR12 testing was more frequent in patients from large, urban, and teaching hospitals, compared to TW (all *P* < 0.01). PCR12 was associated with a higher mean index visit cost (by $97) but lower mean 30-day AGE-related follow-up cost (by $117) than TW. Patients with PCR12 had a lower 30-day AGE-related hospitalization risk than TW (1.7% versus 2.7% *P* < 0.01). Among the 8,451 patients with microbiology data, PCR12 was associated with fewer stool tests per patient (mean 1.61 versus 1.26), faster turnaround time (mean 6.3 versus 25.7 h) and lower likelihood of receiving in-hospital antibiotics (39.4% versus 47.1%, all *P* < 0.01) than TW. A higher percentage of patients with PCR12 had a target pathogen detected (73.1%) compared to PCR < 12 (63.6%) or TW (45.4%, *P* < 0.01). Thus, we found that large multiplex PCR panels were associated with lower 30-day AGE-related follow-up cost and risk of AGE-related hospitalization, and increased diagnostic yield compared to TW.

## INTRODUCTION

Acute infectious gastroenteritis (AGE) is a common reason for visits to U.S. health care providers, primarily in the outpatient setting (1–3). The annual health care cost burden of AGE among hospital-based outpatients alone is estimated to be US $264 million (4). In most cases, AGE is caused by self-limited viral and bacterial infections that do not require diagnostic testing or antibiotic administration (5, 6). However, diagnostic evaluation for AGE is recommended during known or suspected outbreaks, in immunocompromised individuals at increased risk for complications, and for patients at a high risk of spreading the disease to others. In addition, diagnostic stool testing can enable specific directed therapy and provide clinical benefits in patients with dysentery, moderate-severe illness, or symptoms lasting > 7 days (7).

Traditional diagnostic methods (e.g., bacterial culture, antigen testing, microscopy with and without special stains and immunofluorescence) are time-consuming (8) and may be difficult to coordinate due to asynchronous ordering and performance in different laboratory sections or laboratories. Traditional methods also have lower sensitivity, require trained microbiologists to perform (7, 9), and fail to identify a microbial etiology in most cases of AGE (7). For patients in whom diagnostic stool testing is indicated, such limitations may adversely impact health care costs and clinical outcomes– isolation protocols may be inappropriately applied, antibiotic administration may not be timely, and some patients may require additional testing or hospitalization due to delays in diagnosis (10–12).

Multiplex gastrointestinal (GI) polymerase chain reaction (PCR) panels (13) (e.g., bioMérieux BioFire FilmArray GI Panel, Luminex xTAG GI Pathogen Panel, Luminex VERIGENE Enteric Pathogens Test, Applied BioCode GI Pathogen Panel [GPP], BD MAX Enteric Bacterial/Viral/Parasite Panels, Hologic Prodesse ProGastro SSCS Assay) are able to simultaneously detect many of the common agents of AGE, including bacteria, parasites, and viruses (14, 15). Such panels can facilitate accurate and timely identification of AGE pathogens to reduce transmission (16) and appropriately target therapy to shorten the duration of symptoms, prevent complications, and optimize health care resource use (HRU) (17, 18).

Leveraging the largest hospital-based visit database in the U.S., we aimed to examine the relationship between diagnostic test method (traditional stool work- up, multiplex PCR stool panel with < 12 targets, or multiplex PCR stool test with ≥ 12 targets) and HRU and cost in adult outpatients visiting US health care systems for AGE. For a subset of patients with stool microbiology data, we also sought to describe and compare the number and turnaround time of stool tests, commonly detected pathogens, and antibiotic use.

## MATERIALS AND METHODS

### Study design and data source.

A retrospective cohort study was performed using the Premier PINC AI Healthcare Database (PHD). The PHD is a hospital-based, service-level, all-payer discharge database for geographically diverse inpatient and outpatient visits from more than 1,150 U.S. hospitals since 2000 (19, 20). PHD contains information on hospital and visit characteristics, admitting and attending physician specialties, health care payers, and patient data from standard hospital discharge billing files. These data include demographics and disease states, admission and discharge diagnoses, and information on billed services including costs at a departmental level such as medications and devices, laboratory tests performed, diagnostic and therapeutic services, and patient disposition and discharge health status. A subset of hospitals (~ 25%) submit microbiology and laboratory data representing all available data from clinical microbiology or laboratory testing regardless of testing results to the PHD. Microbiology and laboratory data include specimen source, performed tests, and related observations. The PHD has been certified as deidentified and is not considered human subject research. Study data and recorded information cannot be identified directly or through identifiers linked to individuals, and no informed consent was pursued. All data were compliant with the Health Insurance Portability and Accountability Act (HIPAA). As a result of these factors and regulations in the U.S. Title 45 Code of Federal Regulations, Part 46, institutional review board approval for this study was not required. The study followed the Strengthening the Reporting of Observational Studies in Epidemiology (STROBE) (21) reporting guideline.

### Study population.

Adults (≥ 18 years old) with a principal discharge diagnosis of AGE and stool testing performed during an outpatient visit at one of the hospitals submitting data to the PHD between April 1, 2016 and June 30, 2021 were included in the study. AGE was identified using the following International Classification of Diseases, 10th revision, Clinical Modification (ICD-10-CM) diagnosis codes: A00.x-A05.x, A06.0-A06.3, and A07.x-A09.x. Hospitals reported the status of “outpatient visit” based on the Centers for Medicare and Medicaid Services (CMS) definition: Visits lasting less than 2 midnights (22). Emergency department (ED) patients ultimately admitted as inpatients were excluded. If a patient had multiple qualifying visits during the study period, only the first, or “index,” outpatient visit was included in the analysis to represent patient-level data. Stool testing was determined by hospital chargemaster description in itemized billing data during the index visit. Patients were excluded if they had a history of AGE within 30 days prior to the index visit or if the reason for the index visit was for diagnostic testing, same-day surgery, or pre-surgical testing. Patients with standalone *Clostridioides* (*Clostridium*) *difficile* testing were only included if other stool tests were also ordered during the index visit. Patients were included in the subgroup analysis if they had available microbiology data in the PHD for any stool tests performed at the hospital during the index visit.

### Exposure variables.

Types of stool tests were identified by chargemaster descriptions in the itemized billing data during index visit and categorized as: (i) multiplex PCR stool test with < 12 targets (PCR < 12); (ii) multiplex PCR stool test with ≥ 12 targets (PCR ≥ 12); or (iii) traditional stool work-up. Multiplex PCR stool test was defined as having any charges related to a multiplex GI PCR panel. Multiplex PCR ≥ 12 included panels with 12 or more targeted pathogens, and multiplex PCR < 12 included panels with fewer than 12 targeted pathogens. We hypothesized that the outcomes would be different for smaller panels (PCR < 12) and larger panels (PCR ≥ 12) since one of the benefits of multiplex PCR test is testing simultaneously for many different pathogens (23). Traditional stool work-up was defined as having any charges related to stool culture, single pathogen PCR test, immunology test, microscopy, and ova & parasites test during the index visit. Patients with additional traditional stool tests after a multiplex PCR stool test during the index visit were categorized in the multiplex PCR stool test group, as reflexive cultures may be performed following positive PCR results to obtain isolates for species determination, susceptibility testing, and public health surveillance (24).

### Outcome measures.

Main outcomes of interest were HRU and health care cost: (i) discharge status (i.e., discharged home versus requiring post-discharge services including home health, nursing or rehabilitation facility, and hospice); (ii) AGE-related hospitalization and outpatient visits within 30 days of index discharge; (iii) index visit, 30-day AGE-related follow-up, and total index visit plus 30-day AGE-related follow-up costs.

AGE-related hospitalizations and outpatient visits were identified when a patient received inpatient or outpatient care with a principal or secondary discharge diagnosis of AGE within 30 days of index discharge. Index visit, 30-day follow-up, and total index visit plus 30-day follow-up costs included the sum of all costs incurred by the hospital (i.e., room and board, pharmacy, laboratory, imaging, and central supply) during index visit and/or AGE-related hospitalizations and outpatient visits during follow-up (as applicable). In the subgroup analysis, outcomes of interest were number and turnaround time of stool tests, types of identified stool pathogens, and antibiotic use at the hospital during index visit.

### Patient, visit, and hospital characteristics.

Baseline patient characteristics including age, sex, patients’ self-reported race and ethnicity, and hospital characteristics including geographical region (Midwest, Northeast, South, or West), hospital size (number of beds), urbanicity of population served (rural versus urban) and teaching status were provided by participating hospitals.

As a measure of social determinants of health, we used the Social Vulnerability Index (SVI) developed by the Centers for Disease Control and Prevention/Agency for Toxic Substances and Disease Registry (CDC/ATSDR) (25, 26). SVI is county-level data using 15 U.S. census variables, and county of the patient’s residence was used in this study. Overall, SVI is comprised of 4 themes/social factors: socioeconomic status (below poverty; unemployed; income; no high school diploma), household composition and disability (≥ 65 years of age; ≤ 17 years of age; ≥ 5 years of age with a disability; single-parent households), minority status and language (minority; speaks English “less than well”), and housing type and transportation (multi-unit structure; mobile home; crowding; no vehicle; group quarters). The index (percentile rank) ranges from 0 to 1, with higher values representing greater vulnerability.

For baseline comorbidity status, Charlson-Deyo comorbidities were identified using ICD-10-CM diagnosis codes (Table S1) and Charlson-Deyo Comorbidity Index (CCI) score was calculated using a previously validated method. In addition to Charlson-Deyo comorbidities, risk factors for AGE (malnutrition, hypertension, Crohn’s disease, ulcerative colitis, inflammatory bowel disease, irritable bowel syndrome, obesity, hypothyroidism, hemochromatosis or hemoglobinopathy, immunosuppression, and history of transplantation) (16) were identified using ICD-10-CM diagnosis and ICD-10-PCS (procedure) codes (Table S1). Comorbidities and risk factors were assessed during the index outpatient visit and any visit to the same hospital or health system within 180 days prior to the index visit.

### Statistical analysis.

Continuous variables were reported as mean (standard deviation [SD]) or median (1st quartile, 3rd quartile), and categorical variables were reported as counts and percentages. For testing statistical differences between different types of stool test groups (multiplex PCR < 12, multiplex PCR ≥ 12, traditional stool work-up), a Kruskal-Wallis test was used for continuous variables and Pearson’s χ^2^ test was used for categorical variables. To adjust for multiple comparisons, Bonferroni correction was performed for variables when the null hypothesis was rejected. Cost estimates were adjusted to and presented as 2021 US dollars based on Consumer Price Index for urban consumers for hospital and related services.

Unadjusted discharge status (i.e., discharged home) and 30-day risks of AGE-related hospitalization and outpatient visits were calculated as the number and percentage of patients. Adjusted odds were assessed using multivariable logistic regression models with discharge status and 30-day risks of AGE-related hospitalization and outpatient visits as outcomes and types of stool test as predictors (traditional stool work-up was used as the reference group for each model).

Unadjusted means were reported for cost variables by the types of stool tests. Generalized linear regression modeling with gamma or tweedie distribution and loglink function was used, as indicated, to compare the differences in cost between traditional stool work-up group versus multiplex PCR < 12 and multiplex PCR ≥ 12 groups, adjusting for covariates. Covariate-adjusted mean costs were estimated using the recycled prediction method to calculate predicted margins and bootstrapping with 1000 replicates with replacement was used to calculate the confidence intervals (CI).

A priori covariates included patient and hospital characteristics that were significantly different across comparison groups at baseline. Final model covariates were selected using a backward selection method, with a significance level of *P* < 0.10 for covariates to stay in the model and robust standard errors to adjust for clustering of patients within hospitals. Based on the variance inflation factor, covariate multicollinearity was not present in the final models. All analyses and figures were performed and generated using R version 3.6.3 (R Foundation for Statistical Computing).

## RESULTS

Out of 248,896 adult outpatients with a diagnosis of AGE and no history of AGE within 30 days, we identified 36,787 (14.8%) hospital-based AGE outpatients with a multiplex PCR < 12 test (*n* = 4,726), multiplex PCR ≥ 12 test (*n* = 11,098), or traditional stool work-up (*n* = 20,963), as indicated in Fig. S1. Overall, patients had a mean age of 51.2 (SD 20.0) years; more than a quarter of patients were 65 years old or older (Table 1). About 3 out of 5 were women, and 80% were White. The most common health insurance was private insurance (41.4%), followed by Medicare (32.9%). Fewer than 10% of the patients did not have health insurance when they visited the hospital. Most patients visited small (1 to 299 beds, 48.3%), non-teaching (60.5%), and urban (79.4%) hospitals.

**TABLE 1:** Demographics, clinical, and hospital characteristics of acute gastroenteritis outpatients with a stool test, stratified by the type of stool test during index visit

Characteristics	Total		Multiplex PCR < 12		Multiplex PCR ≥ 12		Traditional work-up		Overall
	(N = 36,787)		(N = 4,726)		(N = 11,098)		(N = 20,963)		*P*-value
Patient characteristics									
Age category, in yrs, n (%)									< 0.01
18 to 34	9,707	26.4%	1,297	27.4%	3,272	29.5%	5,138	24.5%	
35 to 49	7,743	21.0%	996	21.1%	2,380	21.4%	4,367	20.8%	
50 to 64	8,703	23.7%	1,078	22.8%	2,504	22.6%	5,121	24.4%	
65 to 74	5,296	14.4%	673	14.2%	1,471	13.3%	3,152	15.0%	
75 +	5,338	14.5%	682	14.4%	1,471	13.3%	3,185	15.2%	
Age, continuous, in yrs									< 0.01
Mean-SD	51.2	20.0	50.9	20.1	49.7	20.1	52.1	19.9	
Median-IQR	51	34, 67	50	33, 67	49	32, 66	53	35, 68	
Sex, n(%)									< 0.01
Female	21,995	59.8%	2,839	60.1%	6,483	58.4%	12,673	60.5%	
Male	14,771	40.2%	1,887	39.9%	4,615	41.6%	8,269	39.4%	
Unknown	21	0.1%	0	0.0%	0	0.0%	21	0.1%	
Race, n(%)									< 0.01
White	29,467	80.1%	3,854	81.5%	8,725	78.6%	16,888	80.6%	
Black	3,355	9.1%	430	9.1%	1,007	9.1%	1,918	9.1%	
Other	3,965	10.8%	442	9.4%	1,366	12.3%	2,157	10.3%	
Ethnicity, n(%)									< 0.01
Hispanic or Latino	4,453	12.1%	484	10.2%	1,812	16.3%	2,157	10.3%	
Not Hispanic or Latino	27,556	74.9%	3,570	75.5%	8,214	74.0%	15,772	75.2%	
Unknown	4,778	13.0%	672	14.2%	1,072	9.7%	3,034	14.5%	
Health insurance status, n (%)									< 0.01
Medicaid	5,608	15.2%	746	15.8%	1,665	15.0%	3,197	15.3%	
Medicare	12,092	32.9%	1,539	32.6%	3,367	30.3%	7,186	34.3%	
Private Insurance	15,237	41.4%	1,915	40.5%	4,754	42.8%	8,568	40.9%	
Uninsured	2,663	7.2%	328	6.9%	942	8.5%	1,393	6.6%	
Other/Unknown	1,187	3.2%	198	4.2%	370	3.3%	619	3.0%	
Social Vulnerability Index (SVI)									
Overall									< 0.01
Mean-SD	0.51	0.24	0.46	0.25	0.52	0.25	0.52	0.24	
Median-IQR	0.53	0.33, 0.69	0.48	0.25, 0.63	0.55	0.38, 0.70	0.54	0.33, 0.69	
Overall category, n (%)									< 0.01
0 to 0.249	6,552	18.4%	1,189	25.2%	2,015	18.4%	3,348	16.9%	
0.250 to 0.499	9,626	27.1%	1,310	27.7%	2,982	27.2%	5,334	26.9%	
0.500 to 0.749	12,669	35.7%	1,574	33.3%	3,950	36.0%	7,145	36.0%	
0.750 to 1.000	6,670	18.8%	652	13.8%	2,017	18.4%	4,001	20.2%	
Hospital characteristics									
Hospital size, n (%)									
1 to 299	17,768	48.3%	2,127	45.0%	4,594	41.4%	11,047	52.7%	< 0.01
300 to 499	10,044	27.3%	1,207	25.5%	3,506	31.6%	5,331	25.4%	
500+	8,941	24.3%	1,392	29.5%	2,998	27.0%	4,551	21.7%	
Unknown	34	0.1%	0	0.0%	0	0.0%	34	0.2%	
Teaching status, n (%)									
Non-teaching	22,256	60.5%	2,911	61.6%	5,929	53.4%	13,416	64.0%	< 0.01
Teaching	14,531	39.5%	1,815	38.4%	5,169	46.6%	7,547	36.0%	
Population served, n (%)									
Rural	7,592	20.6%	833	17.6%	2,043	18.4%	4,716	22.5%	< 0.01
Urban	29,195	79.4%	3,893	82.4%	9,055	81.6%	16,247	77.5%	
Geographic location, n (%)									
Midwest	10,412	28.3%	1,621	34.3%	4,187	37.7%	4,604	22.0%	< 0.01
Northeast	4,060	11.0%	171	3.6%	956	8.6%	2,933	14.0%	
South	17,625	47.9%	2,326	49.2%	4,915	44.3%	10,384	49.5%	
West	4,690	12.7%	608	12.9%	1,040	9.4%	3,042	14.5%	

Use of both multiplex PCR < 12 and multiplex PCR ≥ 12 testing increased over time from 2016 to 2021. While most patients (83%) in 2016 underwent a traditional stool work-up, 34 to 40% of patients in 2019, 2020, and 2021 underwent multiplex PCR ≥ 12 (Table S2 in the Supplement).

Multiplex PCR ≥ 12 patients tended to be younger (mean age 49.7 versus 52.1 years) and were more likely to be men (41.6% versus 39.4%) and Hispanic (16.3% versus 10.3%) compared to patients tested with a traditional stool work-up (all *P* < 0.001). Overall, social vulnerability (based on SVI scores) was similar between patients with multiplex PCR ≥ 12 and traditional stool work-up (mean 0.52 for both, *P* = 0.88); however, multiplex PCR < 12 patients were less likely to be socially vulnerable (i.e., living in counties with higher socioeconomic statuses, household composition and disability, minority status and language, or housing type and transportation) than traditional stool work-up patients (mean 0.46 versus 0.52, *P* < 0.001).

Patients with multiplex PCR ≥ 12 were more likely to visit large (size ≥ 500 beds, 27.0% versus 21.7%), teaching (46.6% versus 36.0%), and urban (81.6% versus 77.5%) hospitals in the Midwest (37.7% versus 22.0%) than patients with traditional stool work-up (all *P* < 0.001). Multiplex PCR ≥ 12 patients were slightly less likely to have a history of all-cause hospitalization within 30 days prior to index visit (3.3% versus 4.1%), hypertension (36.2% versus 39.1%), or hypothyroidism (8.7% versus 9.9%) compared to traditional stool work-up patients (all *P* < 0.001, Table 2), but were more likely to have a history of transplantation (1.4% versus 0.9%) or HIV disease (1.2% versus 0.7%, both *P* < 0.001) than patients with traditional stool work-up. Differences between groups were small, and similar proportions of patients (50.0 versus 49.1%, *P* = 0.10) lacked a known risk factor for AGE at baseline.

**TABLE 2:** Clinical characteristics and health care resource utilization of acute gastroenteritis outpatients with a stool test, stratified by the type of stool test during index visit

Characteristics	Total		Multiplex PCR < 12		Multiplex PCR ≥ 12		Traditional work-up		Overall
	(N = 36,787)		(N = 4,726)		(N = 11,098)		(N = 20,963)		*P*-value
Clinical characteristics									
Prior all-cause hospitalization within 30-days, n (%)	1,416	3.8%	192	4.1%	363	3.3%	861	4.1%	< 0.01
Baseline conditions, n (%)									
Malnutrition	769	2.1%	113	2.4%	211	1.9%	445	2.1%	0.18
Hypertension	14,071	38.2%	1,854	39.2%	4,019	36.2%	8,198	39.1%	<0.01
History of infectious gastroenteritis	733	2.0%	82	1.7%	184	1.7%	467	2.2%	<0.01
Crohn’s disease	417	1.1%	56	1.2%	129	1.2%	232	1.1%	0.65
Ulcerative colitis	546	1.5%	55	1.2%	164	1.5%	327	1.6%	0.04
Inflammatory bowel disease	945	2.6%	111	2.3%	1286	2.6%	548	2.6%	0.30
Other noninfective gastroenteritis and colitis	2,876	7.8%	393	8.3%	866	7.8%	1,617	7.7%	0.16
Irritable bowel syndrome	872	2.4%	128	2.7%	250	2.3%	494	2.4%	0.56
Obesity	3,672	10.0%	486	10.3%	1,219	11.0%	1,967	9.4%	<0.01
Hypothyroidism	3,539	9.6%	492	10.4%	970	8.7%	2,077	9.9%	<0.01
Hemochromatosis or hemoglobinopathy	128	0.3%	11	0.2%	42	0.4%	75	0.4%	0.77
Immunosuppressed	152	0.4%	21	0.4%	52	0.5%	79	0.4%	0.22
History of transplantation	416	1.1%	60	1.3%	160	1.4%	196	0.9%	<0.01
No history of baseline conditions listed above	18,103	49.2%	2,265	47.9%	5,553	50.0%	10,285	49.1%	0.10
Baseline Charlson-Deyo comorbidities, n (%)									
Myocardial infarction	1,754	4.8%	227	4.8%	473	4.3%	1,054	5.0%	< 0.01
Congestive heart failure	2,625	7.1%	342	7.2%	711	6.4%	1,572	7.5%	< 0.01
Peripheral vascular disease	1,539	4.2%	214	4.5%	452	4.1%	873	4.2%	0.69
Cerebrovascular disease	1,662	4.5%	202	4.3%	458	4.1%	1,002	4.8%	0.01
Dementia	965	2.6%	117	2.5%	240	2.2%	608	2.9%	< 0.01
Chronic pulmonary disease	7,206	19.6%	943	20.0%	2,113	19.0%	4,150	19.8%	0.10
Rheumatic disease	901	2.4%	125	2.6%	239	2.2%	537	2.6%	0.02
Peptic ulcer disease	567	1.5%	78	1.7%	170	1.5%	319	1.5%	0.94
Diabetes with chronic complications	3,121	8.5%	446	9.4%	916	8.3%	1,759	8.4%	0.67
Diabetes without chronic complications	4,118	11.2%	502	10.6%	1,186	10.7%	2,430	11.6%	0.01
Hemiplegia or paraplegia	125	0.3%	18	0.4%	32	0.3%	75	0.4%	0.31
Renal disease	3,396	9.2%	437	9.2%	1,004	9.0%	1,955	9.3%	0.41
Mild liver disease	371	1.0%	50	1.1%	96	0.9%	225	1.1%	0.07
Moderate or severe liver disease	314	0.9%	48	1.0%	90	0.8%	176	0.8%	0.79
Any malignancy, including leukemia and lymphoma	2,038	5.5%	243	5.1%	617	5.6%	1,178	5.6%	0.82
Metastatic solid tumor	501	1.4%	67	1.4%	148	1.3%	286	1.4%	0.82
HIV disease	325	0.9%	37	0.8%	137	1.2%	151	0.7%	< 0.01
Charlson Comorbidity Index (CCI) score category, n (%)									
0	20,368	55.4%	2,609	55.2%	6,251	56.3%	11,508	54.9%	0.05
1 to 3	12,121	32.9%	1,565	33.1%	3,569	32.2%	6,987	33.3%	
4+	4,298	11.7%	552	11.7%	1,278	11.5%	2,468	11.8%	
CCI score, continuous									
Mean-SD	1.2	2.0	1.2	2.1	1.2	2.0	1.2	2.0	0.10
Median-IQR	0.0	0.0, 2.0	0.0	0.0, 2.0	0.0	0.0, 2.0	0.0	0.0, 2.0	
Healthcare resource utilizations									
Presence of traditional stool work-up during index visit, n (%)	22,216	60.4%	344	7.3%	909	8.2%	20,963	100.0%	
Outpatient type, n (%)									< 0.01
Clinic	834	2.3%	66	1.4%	456	4.1%	312	1.5%	
Emergency room	33,692	91.6%	4,455	94.3%	10,268	92.5%	18,969	90.5%	
Other	2,261	6.1%	205	4.3%	374	3.4%	1,682	8.0%	
Any abdominal/GI ancillary diagnostic test within 30 days, n (%)	181	0.5%	13	0.3%	38	0.3%	130	0.6%	< 0.01
Discharge status, n (%)									< 0.01
Home	34,884	94.8%	4,503	95.3%	10,698	96.4%	19,683	93.9%	
Home Health	620	1.7%	73	1.5%	131	1.2%	416	2.0%	
Nursing or rehabilitation facility	446	1.2%	60	1.3%	120	1.1%	266	1.3%	
Transferred to another acute care facility	138	0.4%	19	0.4%	8	0.1%	111	0.5%	
Hospice	23	0.1%	2	0.0%	4	0.0%	17	0.1%	
Expired	4	0.0%	0	0.0%	3	0.0%	1	0.0%	
Other/Unknown	672	1.8%	69	1.5%	134	1.2%	469	2.2%	
30-Day risk of AGE-related hospitalization, n (%)	842	2.3%	95	2.0%	184	1.7%	563	2.7%	< 0.01
30-Day risk of AGE-related outpatient visits, n (%)	1,763	4.8%	236	5.0%	526	4.7%	1,001	4.8%	0.89

### Healthcare resource use.

**FIG 1:**
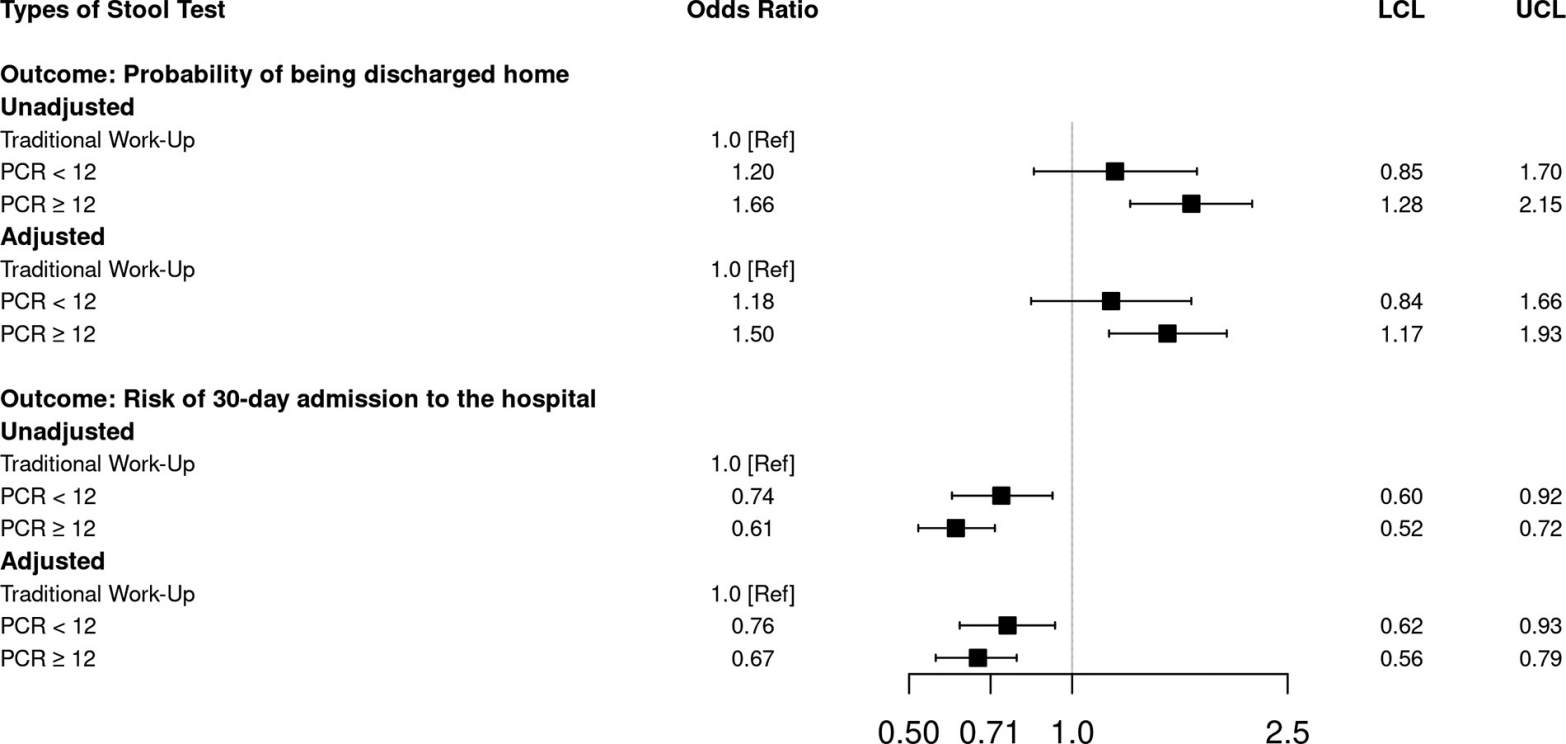
Unadjusted and adjusted associations between outcomes and types of stool tests among acute gastroenteritis outpatients. LCL: lower 95% confidence interval limit. UCL: upper 95% confidence interval limit. PCR < 12: Multiplex PCR stool test with < 12 targets, PCR ≥ 12: multiplex PCR stool test with ≥ 12 targets. Model is adjusted for patient demographic (age group, ethnicity, health insurance status), clinical (prior all-cause hospitalization within 30 days, hypertension, obesity, hypothyroidism, congestive heart failure, dementia) and hospital characteristics (teaching status, urbanicity of population served, geographic location) for ‘probability of being discharged home’ outcome; patient demographic (age group, race, health insurance status), clinical (prior all-cause hospitalization within 30 days, history of acute infectious gastroenteritis, and congestive heart failure) for ‘risk of 30-day admission to the hospital’ outcome.

Most AGE-related outpatient visits occurred in the ED (91.6%, Table 2). Nearly half of the study cohort was seen by an emergency medicine physician (49.1%), followed by hospitalists (15.7%) and internists (15.2%, data not shown). ED utilization was slightly higher among multiplex PCR < 12 (94.3%) and PCR ≥ 12 (92.5%) patients than traditional stool work-up patients (90.5%, both *P* < 0.001). Most (94.8%) patients were discharged home; however, a slightly higher percentage of patients in the multiplex PCR ≥ 12 group was discharged home compared to those in the traditional stool work-up group (96.4% versus 93.9%, *P* < 0.001). After adjusting for patient demographic and clinical, and hospital characteristics, PCR ≥ 12 patients were still 50% more likely to be discharged home than traditional stool work-up patients (adjusted odds ratio [aOR] 1.50, 95% CI 1.17–1.93) (Fig. 1, Table S3).

Overall, 2.3% of patients were hospitalized for AGE within 30 days of the index visit. Compared to multiplex PCR < 12 (2.0%) and PCR ≥ 12 (1.7%) patients, a higher percentage of patients with a traditional work-up were admitted within 30 days (2.7%, both *P* < 0.001, Table 2). After adjusting for patient demographic and clinical characteristics, multiplex PCR ≥ 12 patients were 34% less likely to be admitted within 30 days due to AGE than traditional work-up patients (aOR 0.67, 95% CI 0.56 to 0.79) (Fig. 1, Table S3). Approximately 4.8% of patients had additional outpatient visits due to AGE within 30 days of the index visit, and the percentages were similar for multiplex PCR < 12, multiplex PCR ≥ 12, and traditional work-up patients (overall *P* = 0.89).

### Healthcare cost.

**FIG 2:**
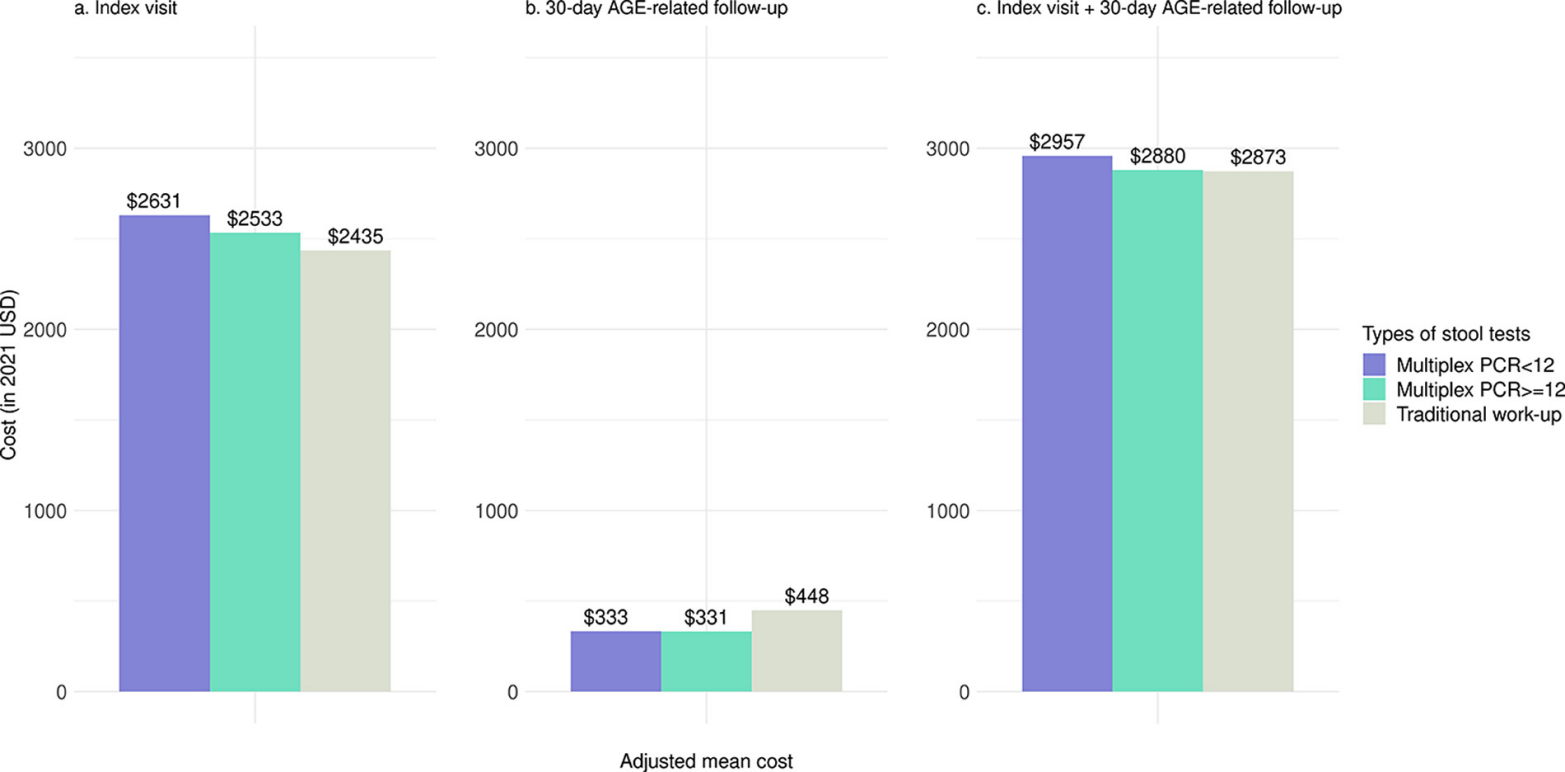
Adjusted mean health care cost of index visit, 30-day AGE-related follow-up, and index visit + 30-day AGE-related follow-up among acute infectious gastroenteritis (AGE) outpatient with a stool test, stratified by the types of stool tests at index visit. PCR < 12: Multiplex PCR stool test with < 12 targets, PCR ≥ 12: multiplex PCR stool test with ≥ 12 targets.

Mean and median costs of the index visit were $2,485 and $1,222, respectively. Mean cost for the index visit was highest for multiplex PCR < 12 ($2,632), followed by multiplex PCR ≥ 12 ($2,529), and traditional stool work-up group ($2,428, all *P* < 0.001) (Table S4). After adjusting for patient, hospital, and clinical characteristics, the mean cost of the index visit for the traditional stool work-up group was $97 lower than that of the multiplex PCR ≥ 12 group and $195 lower than that of multiplex PCR < 12 group (per patient, both *P* < 0.001) (Fig. 2, Table S4). However, traditional stool work-up group had a higher adjusted mean AGE-related cost of 30-day follow-up per patient ($448) compared to the multiplex PCR ≥ 12 group (by $115) and multiplex PCR < 12 group (by $117, both *P* < 0.001). Therefore, the adjusted mean costs of total index plus 30-day AGE-related follow-up visit costs for the multiplex PCR ≥ 12 and multiplex PCR < 12 groups were similar to that of the group with a traditional stool work-up (*P* = 0.90 and *P* = 0.25, respectively).

### Stool test and pathogen results – subgroup analysis.

**FIG 3:**
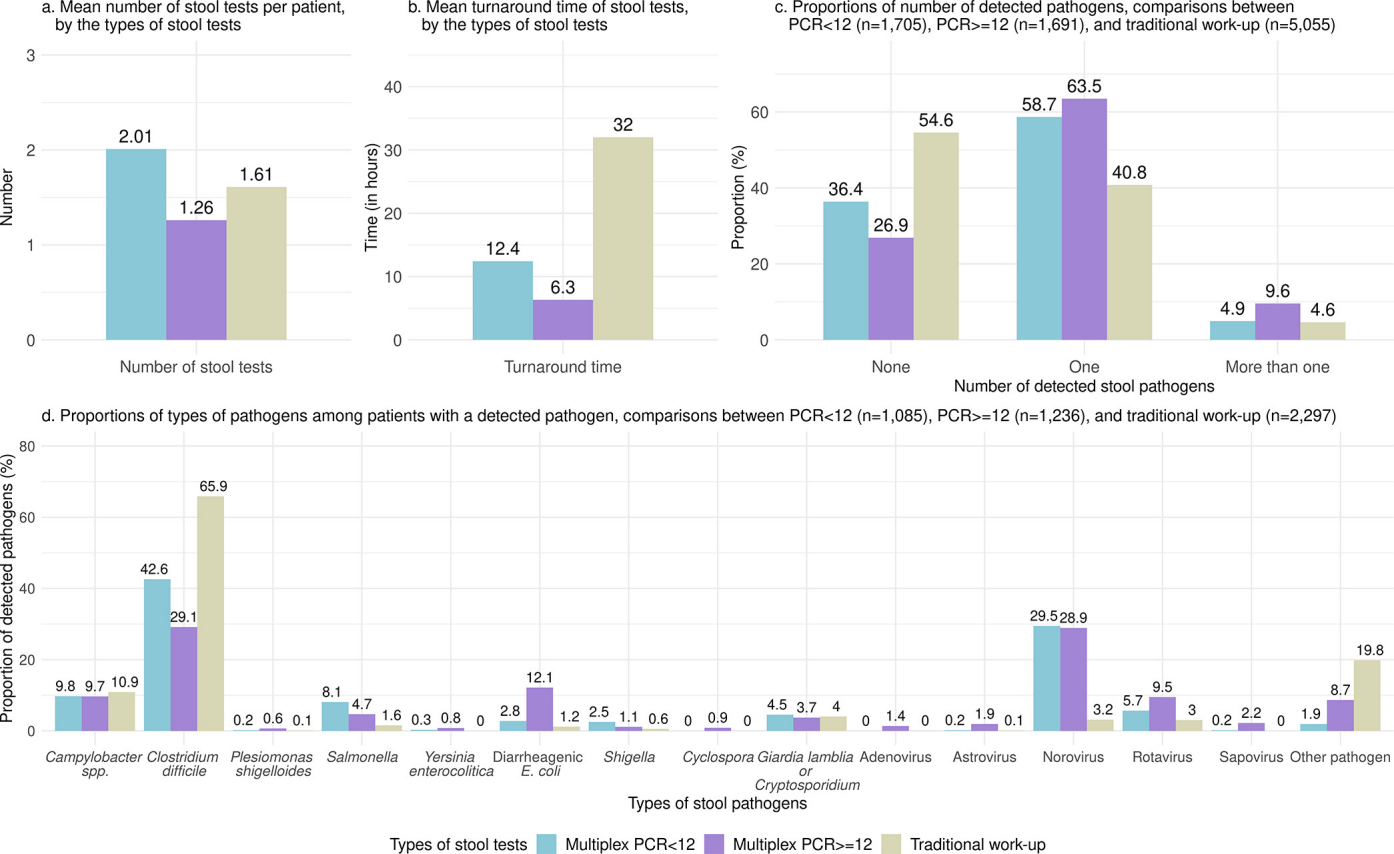
Number of stool tests, turnaround time, and types of detected stool pathogens among hospital-based outpatients who underwent stool testing at the hospital due to acute infectious gastroenteritis. PCR < 12: Multiplex PCR stool test with < 12 targets, PCR ≥ 12: multiplex PCR stool test with ≥ 12 targets.

In our study cohort, microbiology results were available for 8,451 patients (Fig. S1). Among these patients, 20.2% (*n* = 1,705) were tested with multiplex PCR < 12, 20.0% (*n* = 1,691) were tested with multiplex PCR ≥ 12, and 59.8% (*n* = 5,055) underwent a traditional stool work-up. Overall, each patient underwent a mean number of 1.62 (SD 0.95) stool tests during the index visit. Multiplex PCR ≥ 12 patients had the lowest mean number of stool tests (1.26) compared to multiplex PCR < 12 (2.01) and traditional stool work-up (1.61) patients (both *P* < 0.001, Fig. 3). The mean turnaround time for a stool test was the shortest for multiplex PCR ≥ 12 (6.3 h), compared to multiplex PCR < 12 (12.4 h) and traditional stool work-up (32.0 h) (both *P* < 0.001).

Among these 8,451 patients, 45.4% (*n* = 5,922) had no pathogen detected. While only a quarter (26.9%) of multiplex PCR ≥ 12 patients had no pathogen detected, more than half (54.6%) of the traditional stool work-up patients lacked an identifiable etiology (*P* < 0.001). Multiplex PCR ≥ 12 panels were also superior in identifying multiple pathogens (9.6%, *n* = 162), compared to multiplex PCR < 12 panels (4.9%, *n* = 84) or traditional stool work-up (4.6%, *n* = 235, both *P* < 0.001).

Among 4,618 patients with a detected pathogen, the most common pathogens were C. difficile (50.6%), norovirus (16.2%), Campylobacter spp. (*jejuni*, *coli*, and *upsaliensis*, 10.3%), rotavirus (5.4%), Giardia lamblia or *Cryptosporidium* (4.1%), and Salmonella (3.9%). Multiplex PCR ≥ 12 had higher yields of Plesiomonas shigelloides (0.6% versus 0.1%), Salmonella (4.7% versus 1.6%), Shiga toxin-producing Escherichia coli*/*O157 *E. coli* (8.7% versus 1.2%), other diarrheagenic E. coli (enteroaggregative, enteropathogenic, enterotoxigenic, 3.5% versus 0%), *Shigella* (1.1% versus 0.6%), *Cyclospora* (0.9% versus 0%), and enteric viruses (adenovirus [1,4% versus 0%], astrovirus [1.9% versus 0.1%], norovirus [28.9% versus 3.2%], rotavirus [9.5% versus 3.0%], and sapovirus [2.2% versus 0%]), compared to traditional stool work-up (all *P* < 0.001). A higher percentage of patients with traditional stool work-up (47.1%) and multiplex PCR < 12 (48.2%) received antibiotic medications at the hospital in comparison to patients with multiplex PCR ≥ 12 (39.4%, both *P* < 0.001).

## DISCUSSION

This analysis of the largest hospital-based visit database in the U.S. provides novel insights regarding the impact of diagnostic testing method on pathogen detection, health care resource utilization and cost. A recent position paper has called for studies that demonstrate the impact of advanced clinical microbiology technologies at a population level (27). This study represents an effort to leverage the power of big data by analyzing test impact in a large and diverse representative sample that avoids the bias inherent in studies limited to individual institutions or health systems.

Between 2016 and 2021, stool multiplex PCR testing was increasingly utilized for the evaluation of AGE. Patients in larger urban academic hospitals were more likely to undergo multiplex PCR testing than a traditional stool work-up. Multiplex PCR ≥ 12 was more often used in immunocompromised hosts and had a substantially more rapid turnaround time and increased diagnostic yield compared to traditional stool work-up. Although multiplex PCR ≥ 12 was associated with marginally higher health care costs during the index visit, a higher percentage of patients were discharged home and did not require hospitalization within 30 days, resulting in similar overall health care cost for index plus 30-day follow-up visits compared to traditional stool work-up. Furthermore, multiplex PCR ≥ 12 panels were associated with reduced in-hospital administration of antibiotics.

Beal et al. (10) reported that patients with stool samples tested by the FilmArray GI panel had fewer additional stool tests in the ED and inpatient settings. These patients had a shorter length of hospital stay and length of time from stool collection to discharge, suggesting that more rapid identification of the pathogen improved clinical management. Another study has suggested that multiplex PCR ≥ 12 panels have the potential to simplify ordering practices and laboratory workflow (23). Our study also found that stool test turnaround time was significantly shorter (mean 6.3 h) for multiplex PCR ≥ 12 panels compared to multiplex PCR < 12 panels (mean 12.4 h) and traditional stool work-up (mean 32.0 h). Identifying the etiology of AGE in a timely manner is important for outpatients as it is for inpatients because a rapid result can allow appropriately targeted treatment and the withholding of antibiotics when no bacterial pathogen is detected. Patients tested with multiplex PCR ≥ 12 panels had fewer stool tests per patient during the index visit and were more likely to be discharged home and less likely to be hospitalized within 30 days due to AGE.

Using a nationally representative sample, Collins et al. (28) reported that about 13% of ambulatory medical visits due to AGE resulted in an antibiotic prescription, including unnecessarily prescribed antibiotics for viral gastroenteritis and prescription of antibiotics that were not recommended for certain bacterial infections. However, the use of multiplex PCR ≥ 12 panels has been associated with fewer days on antibiotics, and many patients undergoing multiplex PCR ≥ 12 testing have been able to avoid unnecessary additional days of exposure to antibiotics (10). Similarly in our study, we observed that multiplex PCR ≥ 12 patients were less likely to receive antibiotics during the index visit than traditional stool work-up patients.

The spectrum of pathogens detected is comparable to what was observed in smaller studies of selected U.S. outpatient populations (23, 29). Multiplex PCR ≥ 12 panels detected 1 or more pathogens in almost 3 quarters of the patients in this study, while multiple pathogens were detected in fewer than half of patients with a traditional stool work-up. Corroborating the results of earlier studies, multiplex PCR ≥ 12 was superior in detecting enteric viruses and diarrheagenic E. coli compared to a traditional stool work-up and may have contributed to a decrease in antibiotic prescriptions. As other researchers have observed, more work is needed to promote antibiotic stewardship for AGE in outpatient settings. Our observations suggest that accurate and timely diagnosis of AGE with multiplex PCR panels can facilitate these efforts.

An earlier single-center study (10) reported that the use of a multiplex PCR panel was associated with a ~ $300 decrease in health care cost during hospitalization, primarily due to shortened length of stay. In our outpatient study cohort, adjusted mean health care cost of index visit was the highest for multiplex PCR < 12 patients ($2,631), followed by multiplex PCR ≥ 12 patients ($2,533), and traditional work-up patients ($2,435). The cost difference between multiplex PCR ≥ 12 and traditional stool work-up patients was modest (US $97). Moreover, because fewer multiplex PCR ≥ 12 patients incurred AGE-related HRU after the index visit, adjusted mean 30-day AGE-related follow-up cost for patients in this group was lower by US $117 than those with a traditional stool work-up. Adjusted mean total costs for index plus 30-day AGE-related follow-up visit were similar for multiplex PCR ≥ 12 and traditional stool work-up patients, suggesting that use of multiplex PCR ≥ 12 testing does not incur additional AGE-related health care costs.

### Limitations.

This study has several limitations. First, this was a secondary data analysis using a hospital administrative database. AGE status was captured by ICD-10-CM diagnosis codes, and potential coding errors may affect the accuracy of patient identification. Second, type of stool test was identified using chargemaster descriptions, which may have resulted in underreporting. We were also unable to capture tests performed outside of the hospital, which may have underestimated the number of diagnostic tests ordered and the costs of care in the study sample. Third, the incidence of C. difficile and diarrheagenic E. coli may be underreported because some laboratories do not report these targets on multiplex PCR panels (30). Fourth, the study cohort did not include children and adolescents (< 18 years), and it should be acknowledged that the burden of many viral causes of AGE (e.g., norovirus, astrovirus, sapovirus, rotavirus, enteric adenovirus) is highest among children < 5 years of age. Fifth, the findings in this study were only applicable to 15% of adult outpatients with a diagnosis of AGE in whom the decision to perform a stool test had already been made. Overall efficiency of multiplex PCR tests will be dependent upon the decision to test. The adoption of large multiple PCR panels along with an increase in testing will not necessarily result in greater efficiencies. Furthermore, while our study showed that multiplex PCR ≥ 12 panels detected more targets, false-positive results may also occur (e.g., Vibrio cholerae, Entamoeba histolytica) (31, 32), and cultures may still be required for antimicrobial susceptibility testing, public health surveillance, and outbreak investigation (24). Finally, the study cohort was limited to hospital-based outpatients who were primarily evaluated in the ED setting; however, we did not include ED patients who were ultimately admitted as inpatients and therefore believe our findings are more generalizable to other outpatient settings than overall ED setting. Additional research is needed to understand the extent of generalizability of our results to the overall outpatient population.

### Conclusions.

This is one of the few studies to assess the relationship between diagnostic test method and health care resource use and cost of acute infectious gastroenteritis patients in a large U.S. hospital-based outpatient cohort. Multiplex PCR GI panels with 12 or more targets were associated with fewer stool tests per patient, more rapid turnaround time, higher rates of pathogen detection, and reduced in-hospital administration of antibiotics. Although the likelihood of additional inpatient and outpatient visits due to AGE within 30 days of discharge was low overall, multiplex PCR ≥ 12 panels were associated with a further reduction in 30-day risk of AGE-related hospitalization. Higher material costs were offset by lower costs of follow-up care, so that overall health care costs for the index visit plus 30-day follow-up were comparable for PCR panels and traditional work-up. These results may help clinicians and administrators responsible for the hospital-based diagnosis and care of adult outpatients with AGE to provide better care for these patients. Further studies may be needed to assess the utility and impact of stool diagnostic testing in other ambulatory care settings.
