# Supplementary material for: Relationship between Diagnostic Method and Pathogen Detection, Healthcare Resource Use, and Cost in U.S. Adult Outpatients Treated for Acute Infectious Gastroenteritis
Source: J Clin Microbiol. 2023 Jan 16;61(2):e01628-22. doi: 10.1128/jcm.01628-22 (PMC9945572; doi:10.1128/jcm.01628-22)

Supplement

Manuscript Title: Relationship Between Diagnostic Method and Pathogen Detection, Healthcare Resource Use, and Cost in U.S. Adult Outpatients Treated for Acute Infectious Gastroenteritis

Authors: Rena C. Moon, Tammy C. Bleak, Ning A. Rosenthal, Brianne Couturier, Rachael Hemmert, Tristan T. Timbrook, Harold Brown, Ferric C. Fang

Table of Content

eTable 1 ………………………………………………………………… page 2

eTable 2 ………………………………………………………………… page 4

eTable 3 ………………………………………………………………… page 5

eTable 4 ………………………………………………………………… page 6

eFigure 1 ………………………………………………………………… page 7

Supplement eTable 1. ICD-10-CM diagnosis and procedure codes for Charlson-Deyo comorbidities and risk factors for infectious gastroenteritis

Comorbidity and Conditions	Type	ICD-10-CM codes
Myocardial infarction	Dx	I21.x, I22.x, I23.x, I25.2
Congestive heart failure	Dx	I50.x
Peripheral vascular disease	Dx	I73.9, I71.00, I71.01, I71.02, I71.03, I71.1, I71.2, I71.3, I71.4, I71.5, I71.6, I71.8, I71.9, I96, Z95.828
	Proc	04RK07Z, 04RK0JZ, 04RK0KZ, 04RK47Z, 04RK4JZ, 04RK4KZ, 04RL07Z, 04RL0JZ, 04RL0KZ, 04RL47Z, 04RL4JZ, 04RL4KZ, 04RM07Z, 04RM0JZ, 04RM0KZ, 04RM47Z, 04RM4JZ, 04RM4KZ, 04RN07Z, 04RN0JZ, 04RN0KZ, 04RN47Z, 04RN4JZ, 04RN4KZ, 04RP07Z, 04RP0JZ, 04RP0KZ, 04RP47Z, 04RP4JZ, 04RP4KZ, 04RQ07Z, 04RQ0JZ, 04RQ0KZ, 04RQ47Z, 04RQ4JZ, 04RQ4KZ, 04RR07Z, 04RR0JZ, 04RR0KZ, 04RR47Z, 04RR4JZ, 04RR4KZ, 04RS07Z, 04RS0JZ, 04RS0KZ, 04RS47Z, 04RS4JZ, 04RS4KZ, 04RT07Z, 04RT0JZ, 04RT0KZ, 04RT47Z, 04RT4JZ, 04RT4KZ, 04RU07Z, 04RU0JZ, 04RU0KZ, 04RU47Z, 04RU4JZ, 04RU4KZ, 04RV07Z, 04RV0JZ, 04RV0KZ, 04RV47Z, 04RV4JZ, 04RV4KZ, 04RW07Z, 04RW0JZ, 04RW0KZ, 04RW47Z, 04RW4JZ, 04RW4KZ, 04RY07Z, 04RY0JZ, 04RY0KZ, 04RY47Z, 04RY4JZ, 04RY4KZ
Cerebrovascular disease	Dx	I60.x, I61.x, I62.x, I63.x, I65.x, I66.x, I67.x, I68.x, I69.x, G45.x
Dementia	Dx	F03.90, F01.50, F01.51, F03.91, F02.80, F02.81
Chronic pulmonary disease	Dx	J40, J41.0, J41.1, J44.9, J44.0, J41.8, J42, J43.9, J45.20, J45.21, J45.22, J44.1, J45.990, J45.991, J45.909, J45.998, J45.902, J45.901, J47.9, J47.1, J67.0, J67.1, J67.2, J67.3, J67.4, J67.5, J67.6, J67.7, J67.8, J67.9, J60, J61, J62.8, J63.0, J63.1, J63.2, J63.3, J63.4, J63.5, J63.6, J66.0, J66.1, J66.2, J66.8, J64, J68.4
Rheumatic disease	Dx	M32.10, M34.0, M34.1, M34.9, M33.20, M06.9, M05.00, M05.30, M05.60, M06.1, M05.10, M35.3
Peptic ulcer disease	Dx	K25.0, K25.1, K25.2, K25.3, K25.4, K25.5, K25.6, K25.7, K25.9, K26.0, K26.1, K26.2, K26.3, K26.4, K26.5, K26.6, K26.7, K26.9, K27.0, K27.1, K27.2, K27.3, K27.4, K27.5, K27.6, K27.7, K27.9, K28.0, K28.1, K28.2, K28.3, K28.4, K28.5, K28.6, K28.7, K28.9
Mild liver disease	Dx	K70.30, K73.9, K73.0, K75.4, K73.2, K73.8, K74.0, K74.60, K74.69, K74.3, K74.4, K74.5
Diabetes without chronic complication	Dx	E11.9, E10.9, E13.9, E11.65, E10.65, E13.65, E10.1x, E11.1x, E13.1x, E11.0x, E13.0x, E11.64x, E10.64x, E13.64x
Diabetes with chronic complication	Dx	E10.2x, E10.3x, E10.4x, E10.5x, E10.61x, E10.62x, E10.63x, E10.69, E10.8, E11.2x, E11.3x, E11.4x, E11.5x, E11.61x, E11.62x, E11.63x, E11.69, E11.8, E13.2x, E13.3x, E13.4x, E13.5x, E13.61x, E13.62x, E13.63x, E13.69, E13.8
Hemiplegia or paraplegia	Dx	G04.1, G11.4, G80.1, G80.2, G81.x, G82.x, G83.0, G83.1x, G83.2x, G83.3x, G83.4, G83.9
Moderate or severe renal disease	Dx	I12.0, I13.11, I13.2, N03.2, N03.3, N03.5, N03.8, N03.9, N05.2 N05.5, N05.9, N08, N18.x, N19.x, N25.x, Z49.0x, Z49.3x, Z94.0, Z99.2

Any malignancy, including lymphoma and leukemia, except malignant neoplasm of skin	Dx	C00.x-C75.x (except C43.x and C44.x), C81.x-C85.x, C88.x, C90.x, C91.x-C95.x, C96.x, C7A.xxx, C7B.xxx, D00.xx, D01.xx, D02.x, D03.xx, D05.xx, D06.x, D07.xx, D09.xx, D47.9
Moderate or severe liver disease	Dx	I85.00, I85.01, I85.10, I85.11, I86.4, K70.4x, K71.1x, K71.7, K72.xx, K76.6, K76.7
Metastatic solid tumor	Dx	C77.x, C78.x, C79.x, C80.0
HIV disease	Dx	B20
Malnutrition	Dx	E40, E41, E42, E43, E44.x, E45, K90.x, E64.0, D50, D51.x, D52.x, D53.x, T73.0, R64
Hypertension	Dx	I10, I11.x, I12.x, I13.x, I15.x, I16.x
Crohn's disease	Dx	K50.x
Ulcerative colitis	Dx	K51.x
Inflammatory bowel disease	Dx	K50.x, K51.x, K52.3
Other noninfective gastroenteritis and colitis	Dx	K52.0, K52.1, K52.2x, K52.8x, K52.9
Irritable bowel syndrome	Dx	K58.x
Obesity	Dx	E66.0x, E66.1, E66.2, E66.8, E66.9, Z68.3x, Z68.4x
Hypothyroidism	Dx	E00.x, E01.x, E02.x, E03.x, E89.0
Hemochromatosis or hemoglobinopathy	Dx	E83.11x, D56.x, D57.x, D58.x, D59.x
Immunosuppression	Dx	D70.0, D70.1, D70.2, D70.3, D70.4, D70.8, D70.9, D71, D80.x, D81.x, D82.x, D83.x, D84.x, D89.x, T86.x, Z94.x,
	Proc	0BY%, 02YA%, 0DY5%, 0DY6%, 0DY8%, 0DYE%, 0FY0%, 0FYG%
History of transplantation	Dx	T86%, Z94%, D89.813
	Proc	0BY%, 02YA%, 0DY5%, 0DY6%, 0DY8%, 0DYE%, 0FY0%, 0FYG%

ICD-10-CM: International Classification of Diseases, 10th revision, Clinical Modification
Dx: diagnosis
Proc: procedure
HIV: human immunodeficiency virus

Supplement eTable 2. Types of stool tests among acute infectious gastroenteritis outpatients by year

Study Period	Total		Multiplex PCR < 12		Multiplex PCR ≥ 12		Traditional work-up	
	(N = 36,787)		(N = 4,726)		(N = 11,098)		(N = 20,963)	
2016 (Apr 1 – Dec 31)	4,909	100%	283	5.8%	541	11.0%	4,085	83.2%
2017 (Jan 1 – Dec 31)	7,866	100%	714	9.1%	1,788	22.7%	5,364	68.2%
2018 (Jan 1 – Dec 31)	8,054	100%	773	9.6%	2,722	33.8%	4,559	56.6%
2019 (Jan 1 – Dec 31)	9,171	100%	1,377	15.0%	3,727	40.6%	4,067	44.3%
2020 (Jan 1 – Dec 31)	4,601	100%	1,021	22.2%	1,577	34.3%	2,003	43.5%
2021 (Jan 1 – Jun 30)	2,186	100%	558	25.5%	743	34.0%	885	40.5%

Supplement eTable 3. Unadjusted and adjusted associations between outcomes and types of stool tests among acute infectious gastroenteritis outpatients

**Model Specification**	**Unadjusted**		**Adjusted**					
	**Model 1**		**Model 2[a]**		**Model 3[b]**		**Model 4[c,d]**	
**Types of Stool Tests**	**OR**	**95% CI**	**OR**	**95% CI**	**OR**	**95% CI**	**OR**	**95% CI**
Outcome: Probability of being discharged home								
Traditional Work-Up	1 [Ref]	[Ref]	1 [Ref]	[Ref]	1 [Ref]	[Ref]	1 [Ref]	[Ref]
PCR < 12	1.20	0.85-1.70	1.17	0.84-1.63	1.18	0.86-1.60	1.18	0.84-1.66
PCR ≥ 12	**1.66**	1.28-2.15	**1.53**	1.17-2.00	**1.54**	1.21-1.97	**1.50**	1.17-1.93
Outcome: Risk of 30-day AGE-related admission to the hospital								
Traditional Work-Up	1 [Ref]	[Ref]	1 [Ref]	[Ref]	1 [Ref]	[Ref]	1 [Ref]	[Ref]
PCR < 12	**0.74**	0.60-0.92	**0.76**	0.62-0.94	**0.76**	0.62-0.94	**0.76**	0.62-0.93
PCR ≥ 12	**0.61**	0.52-0.72	**0.66**	0.56-0.78	**0.67**	0.56-0.79	**0.67**	0.56-0.79

[a] Model 2 is adjusted for sex, age group, race, and ethnicity.
[b] Model 3 is adjusted for sex, age group, race, ethnicity, health insurance status and hospital characteristics (size, teaching status, urbanicity of population served, and geographic location)
[c] Model 4 is adjusted for patient demographic (age group, ethnicity, health insurance status), clinical (prior all-cause hospitalization within 30 days, hypertension, obesity, hypothyroidism, congestive heart failure, dementia) and hospital characteristics (teaching status, urbanicity of population served, geographic location) for 'probability of being discharged home' outcome; patient demographic (age group, race, health insurance status), clinical (prior all-cause hospitalization within 30 days, history of acute infectious gastroenteritis, and congestive heart failure) for 'risk of 30-day admission to the hospital' outcome.
[d] Models 4 are final models for both outcomes.

Supplement eTable 4. Unadjusted and adjusted healthcare costs during index visit, 30-day AGE-related follow-up, and index visit plus 30-day AGE-related follow-up among acute gastroenteritis outpatients with a stool test, stratified by the types of stool tests at index visit

	**Multiplex PCR < 12 (N = 4,726)**	**Multiplex PCR ≥ 12 (N = 11,098)**	**Traditional work-up (N = 20,963)**	***Multiplex PCR<12 vs. Traditional Work-up***	***Multiplex PCR≥12 vs. Traditional Work-up***
Unadjusted Results (in 2021 US dollars)				***p-value***	***p-value***
**Index visit cost**				< .001	< .001
Mean (std)	$2,632 ($3,706)	$2,529 ($3,399)	$2,428 ($3,298)		
**30-day AGE-related follow-up cost**				< .001	< .001
Mean (std)	$298 ($2,316)	$323 ($3,573)	$453 ($3,392)		
**Total cost for index visit and 30-day AGE-related follow-up**				< .001	< .001
Mean (std)	$2,930 ($4,417)	$2,852 ($5,054)	$2,881 ($4,823)		
Median (q1, q3)	$1,407 ($735, $3,728)	$1,322 ($733, $3,510)	$1,266 ($666, $3,544)		
Adjusted Results (in 2021 US dollars)				***Difference***	***Difference***
**Index visit cost**					
Adjusted Mean	$2,631	$2,533	$2,435	$195	$97
95% Confidence Interval	$2,624 - $2,639	$2,527 - $2,541	$2,429 - $2,442	$195 - $196	$97 - $98
**30-day AGE-related follow-up cost**					
Adjusted Mean	$333	$331	$448	($115)	($117)
95% Confidence Interval	$$329 - $336	$328 - $334	$444 - $452	($116) - ($114)	($118) – ($116)
**Total cost for index visit and 30-day AGE-related follow-up**					
Adjusted Mean	$2,957	$2,880	$2,873	$83	$7
95% Confidence Interval	$2,947 - $2,968	$2870 - $2,890	$2,864 - $2,885	$83 - $84	$6 - $7

*All adjusted models were adjusted for sex, age group, race, ethnicity, health insurance status, hospital size, hospital teaching status, urbanicity of population served, geographic location, and Charlson comorbidity index category.
*Index visit cost and total cost models were adjusted using GLM regression with gamma variance and log-link function.
*Follow-up visit cost model was adjusted using GLM regression with tweedie distribution and log-link function.
*All cost and models were estimated using recycled prediction method and bootstrapping (n = 1,000 simulations) for the 95% CI

Supplement eFigure 1. Patient selection flowchart

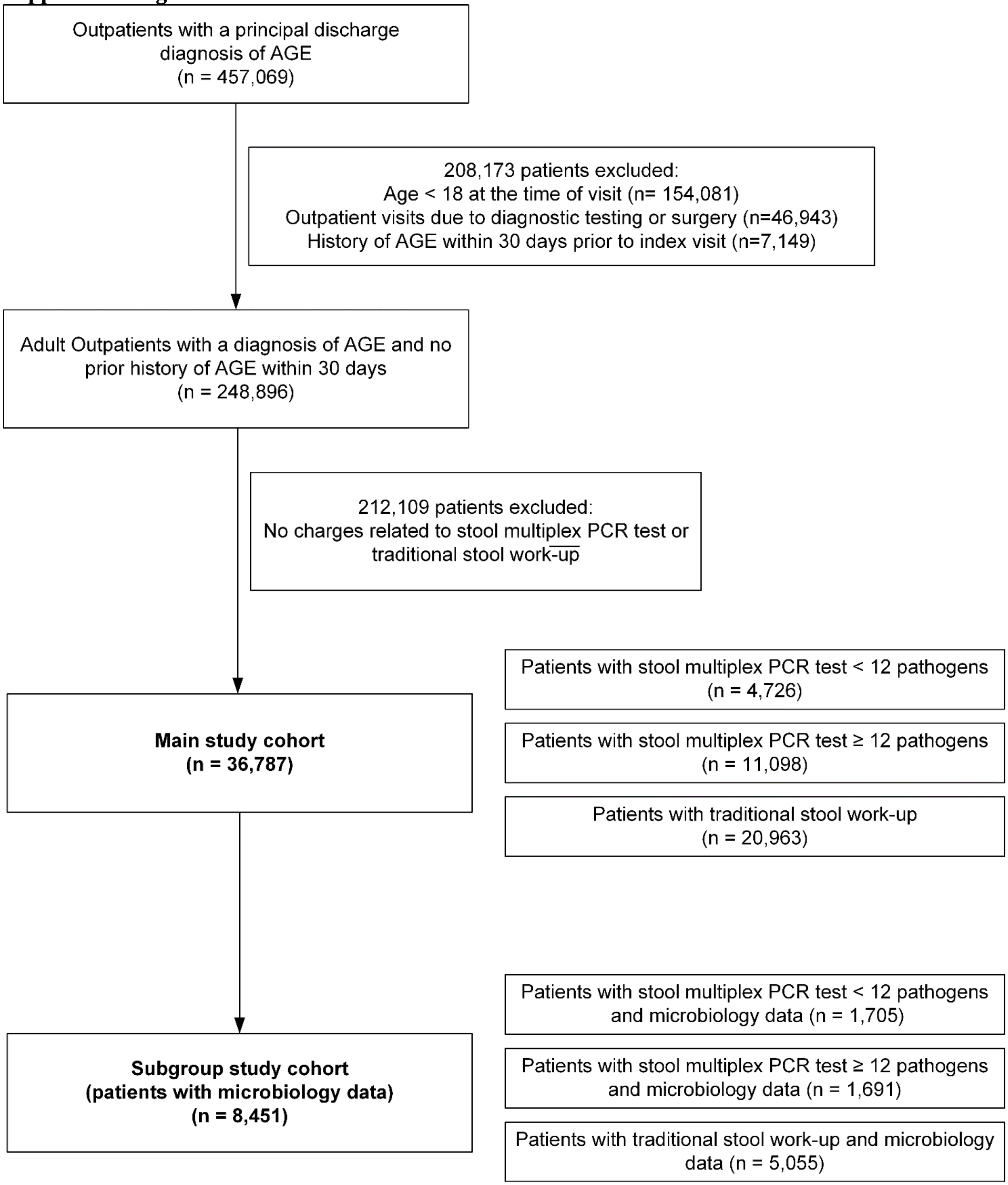

Supplement: Supplemental file 1 — Tables S1 to S4 and Fig. S1. Download jcm.01628-22-s0001.pdf, PDF file, 0.3 MB [file jcm.01628-22-s0001.pdf]
